# Blocking Superantigen-Mediated Diseases: Challenges and Future Trends

**DOI:** 10.1155/2024/2313062

**Published:** 2024-01-17

**Authors:** Pengbo Wang, Zina Fredj, Hongyong Zhang, Guoguang Rong, Sumin Bian, Mohamad Sawan

**Affiliations:** CenBRAIN Neurotech, School of Engineering, Westlake University, Hangzhou 310030, China

## Abstract

Superantigens are virulence factors secreted by microorganisms that can cause various immune diseases, such as overactivating the immune system, resulting in cytokine storms, rheumatoid arthritis, and multiple sclerosis. Some studies have demonstrated that superantigens do not require intracellular processing and instated bind as intact proteins to the antigen-binding groove of major histocompatibility complex II on antigen-presenting cells, resulting in the activation of T cells with different T-cell receptor V*β* and subsequent overstimulation. To combat superantigen-mediated diseases, researchers have employed different approaches, such as antibodies and simulated peptides. However, due to the complex nature of superantigens, these approaches have not been entirely successful in achieving optimal therapeutic outcomes. CD28 interacts with members of the B7 molecule family to activate T cells. Its mimicking peptide has been suggested as a potential candidate to block superantigens, but it can lead to reduced T-cell activity while increasing the host's infection risk. Thus, this review focuses on the use of drug delivery methods to accurately target and block superantigens, while reducing the adverse effects associated with CD28 mimic peptides. We believe that this method has the potential to provide an effective and safe therapeutic strategy for superantigen-mediated diseases.

## 1. Introduction

Pathogenic microorganisms are a class of pathogens that pose a significant threat to animals. These pathogens possess multifaceted capabilities, including the production of various morbigenous elements such as surface-expressed matrix binding proteins (e.g., fibronectin-binding proteins), immune inhibitors (e.g., chemotaxis inhibitory protein of *Staphylococcus aureus*, the chemotaxis inhibitory protein of staphylococci), cytolytic toxins (e.g., *α*-toxin and leucocidins), and superantigens (SAgs) [1]. The presence of microorganisms and their associated elements can activate both innate and acquired adaptive responses, leading to immune dysregulation in the host and resulting in a range of diseases. The term “superantigen” was introduced by White et al. [2]. SAgs have a tendency to bind major histocompatibility complex class II molecules without antigen processing and the T-cell receptor (TCR) V*β*-chain, resulting in the formation of an unconventional and massive T-cell activation complex [3].

SAgs are a unique class of protein molecules that have the ability to activate a large number of T cells, leading to extensive immune reactions and systemic inflammatory responses. These molecules can originate from various microorganisms such as bacteria and viruses and are expressed in human tumor cells and other human cells. Based on their sources and structural features, SAgs can be classified into several categories, including (1) bacterial SAgs, (2) viral SAgs, and (3) human endogenous retroviral SAgs.

SAg-mediated diseases can be classified into two categories: acute diseases and chronic conditions that result from immune system dysregulation. Due to their short generation time, bacteria can evolve rapidly to produce different types of SAgs, making it challenging for the slowly evolving immune system to develop effective antibodies against them. Additionally, SAgs can induce anergy in mature T cells and promote the deletion of developing T cells, further impeding antibody production. On the one hand, bacteria invade the host to release a variety of SAgs, and the pathogenic mechanism is complex and variable. Thus, the development of antibodies to block SAgs faces great challenges. The complex and heterogeneous pathogenic mechanisms employed by bacteria to release SAgs contribute to the difficulty in developing antibodies to block them. Notably, most SAgs bind exclusively to the *α* or *β* binding sites of MHC II molecules and activate a limited number of TCR V*β* cells. Therefore, developing antibodies that target SAgs at these two sites may be a promising strategy. For viral SAgs, genes encoding a single SAg, such as MMTV, can serve as a template for the development of antibodies against SAgs. Alternatively, the overactivation of the immune system caused by SAgs has been exploited for tumor therapy, and numerous studies have demonstrated the effectiveness of SAg fusion proteins in treating cancer [4, 5].

In this review, we present a comprehensive analysis of the current state of research on SAgs, focusing on their classification and structural characteristics. Additionally, the immune system's response to SAgs and various methods for blocking their activity, such as drug delivery methods, are discussed along with the different applications of SAgs, as well as prospects for future development directions.

## 2. SAgs Classifications

### 2.1. Bacterial SAgs

Bacterial SAgs are the earliest discovered type of SAgs, originating from a variety of pathogenic bacteria such as *S. aureus* [6] and Streptococcus [7]. These bacterial SAgs are typically single protein molecules with unique structural features that enable tight binding to TCRs and MHC molecules, resulting in extensive T-cell activation and secretion of inflammatory factors. The most extensively studied SAgs are those secreted by Staphylococcus and Streptococcus, with 26 staphylococcal and 11 streptococcal SAgs identified to date. They are the most potent T-cell mitogens known [8, 9]. The *S. aureus* SAgs include toxic shock syndrome toxin 1 (TSST-1), staphylococcal enterotoxins (SEs) A-E, G-I, and SE-like (SEl) SAgs J-Z [9, 10]. However, unlike SEs, SEL proteins cannot induce emesis or activate T cells [11]. Streptococcal SAgs include streptococcal pyrogenic exotoxin (Spe) A, SpeC, SpeG, SpeH, SpeI, SpeJ, SpeK, SpeL, SpeM and SSA. Bacterial SAgs are typically classified into five distinct groups based on their structure. Among these, staphylococcal SAgs fall under Groups I–III and V, where group IV is composed entirely of related streptococcal SAgs. This grouping strategy reflects certain SAG characteristics, such as MHC II binding modes and, in some cases, disease associations [11, 12].

### 2.2. Amino Acid Sequence Analysis of Bacterial SAgs

**Figure 1:**
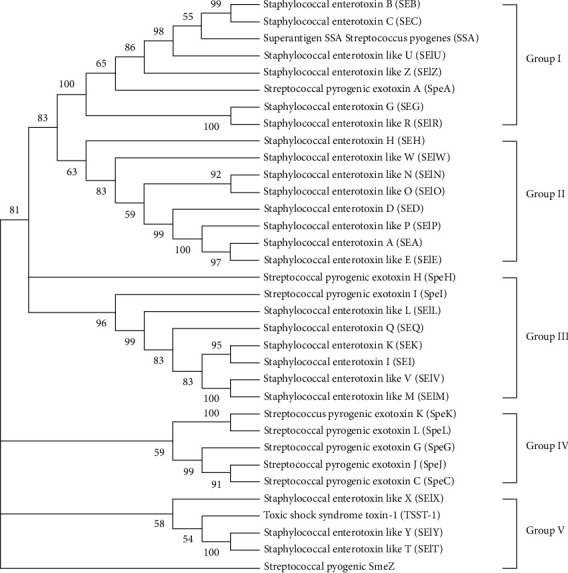
Phylogenetic tree of known bacterial SAgs: the unrooted tree was constructed using the amino acid sequence alignment method of unweighted pair group using arithmetic averages (UPGMA) in Mega11. It is divided into five major groups, representing distinct classes of SAgs.

Although bacterial SAgs vary in their amino acid sequences, they all overactivate the immune system. This suggests that they may be evolutionarily related. Bacterial SAgs can be classified based on their structure and source or origin, as illustrated in Figure 1.

### 2.3. The Structure of Bacterial SAgs

Almost all SAgs are characterized by a specific structural arrangement, which includes an *α*-helix at the N-terminus and a *β*-fold at the C-terminus. Additionally, SAgs possess a conserved domain for a 12-peptide. The N-terminal domain is a mixed *β*-barrel with a Greek-key topology, also known as an oligonucleotide OB-fold [13, 14]. Although SAgs share similar folds, they differ primarily in highly variable regions composed of several surface loops, including the disulfide-bonded loop (which is absent from TSST-1) and a region at the amino terminus. Therefore, understanding these structural features is crucial in the development of novel therapies to combat diseases associated with excessive immune activation, such as TSS and autoimmune disorders.

### 2.4. Viral SAgs

Viral SAgs are mainly produced by virus-infected cells, such as HIV [15], Epstein–Barr virus (EBV) [16], mouse hepatitis virus (MHV) [17], and human endogenous retrovirus (HERV) SAgs. Unlike bacterial SAgs, viral SAgs are typically complex protein structures that are composed of multiple open-reading frames within the viral genome. Despite this difference, the structure and mechanism of action of viral SAgs are similar to those of bacterial SAgs.

Numerous viral SAgs have been identified, including the nucleocapsid of rabies virus [18], HIV-gp120 [15], and MMTV-encoded Mls [19]. Additionally, two types of cytomegalovirus tumor virus [20] have been found to possess superantigen activity. However, it is worth noting that HIV gp120 preferentially activates VH3^+^ B cells, and this activation leads to significant loss of VH3^+^ B cells late in infection. This suggests that HIV-gp120 is a B-cell superantigen [15]. The SAg binding site of HIV-gp120 consists of two discrete regions of the protein sequence. The core motif is a discontinuous epitope spanning the amino-terminal region on both sides of the V4 variable domain and the C4 constant domain. The C2 structure and residues also appear to play a supporting role in the SAgs domain of Igs in the gp120 domain [21].

Similar to bacterial SAgs, EBV SAgs can activate T cells. However, EBVs do not contain genes that encode the SAs themselves. Instead, EBVs activate T-cell proliferation in an indirect manner. In 1996, researchers discovered that EBVs specifically activated TCR V*β*13, but they were unable to isolate the SAg encoded by EBVs [16]. Further studies have revealed that EBV infection activates endogenous superantigen genes in the human genome, such as HERV-K18, which encodes the env protein, and SAg that specifically stimulates TCR V*β*13 and TCR V*β*9 proliferation [22]. The expression of the HERV gene may be related to the activation of the inflammatory response [23, 24].

In 1997, Conrad et al. [25] made a groundbreaking discovery by uncovering the first evidence of a possible endogenous SAg. Their study on type I diabetes revealed that a significant proportion of V*β*7 T cells infiltrated the pancreatic islets in several patients.

Subsequent studies have shown that IDDMK_1,2_ is just one of several variants of HERV-K10. However, IDDMK_1,2_ is not considered a functional virus, according to this study [26]. Further research suggests that endogenous SAg is encoded by the env gene of a HERV. Initially named the IDDMK_1,2_ 22 allele, it was later identified to be identical to HERV-K18 [27]. This SAg demonstrated specificity toward V*β*7 T cells.

Plasma samples from newly diagnosed IDDM patients were found to contain viral RNA, although subsequent studies were unable to reproduce these findings [26, 28–30]. This difference may be due to individual differences in patients and different factors causing the disease. However, the discovery of HERV-K18 located within the first intron of CD48 on chromosome 1 has opened up the possibility of identifying superantigen RNA [27]. In this context, Huber's group had previously conducted investigations into SAg-like activity associated with EBV infection [16]. EBV-infected B cells induce CD48 expression, and an upstream EBV-inducible enhancer has been identified. Subsequent research showed that the previously described EBV-related SAg activity was encoded by alleles of the HERV-K18 env gene [22]. The more common K18.1 and K18.2 alleles were both found to encode SAg genes specific for TCR V*β*13 and V*β*9. Transcriptional activation of HERV-K18 env occurred following EBV infection, and EBV-associated SAg activity was inhibited with an antiserum to HERVK18 env. In another paper from Conrad's group [24], three alleles of HERV-K18 env were identified and distinguished from other KERV-K provirus genes based on their insertion site within the CD48 intron, an achievement that was essential for this work [25]. All three had SAg activity and primarily stimulated V*β*7 (and possibly V*β*13.1) T cells. Thus, the SAg activity appears to be contained within the N-terminal of approximately 150 amino acids. The expression of the HERV-K18 alleles was strongly induced by *α*IFN treatment of PBLs, and V*β*7-specific SAg activity was inhibited with specific antisera to env peptides. Induction of HERV-K18 RNA by *α*IFN occurred in CD2- cells (which include B cells) but not in CD2^+^ cells.

## 3. Biological Effects of SAs

### 3.1. MHC Ⅱ Binding

**Figure 2:**
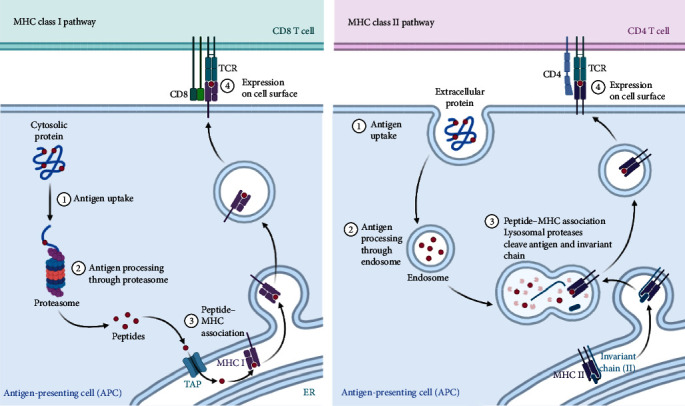
Mechanism by which antigens activate the immune system: endogenous antigens are processed by proteasome into antigenic peptides, which recruit MHC I to the endoplasmic reticulum and bind to it to form the antigenic peptide-MHC I complex. The complex is processed on the Golgi apparatus/endoplasmic reticulum and secreted to the cell membrane surface, and the antigen is presented to CD8 T cells. After processing by lysosomes into antigenic peptides, exogenous antigens recruit MHC II on the endoplasmic reticulum and bind to it to form an antigenic peptide-MHC II complex. After processing by the Golgi apparatus/endoplasmic reticulum, the antigen is secreted to the surface of the cell membrane and presented to CD4 T cells.

Conventional antigens are typically processed into small peptides within the lysosomal compartments of antigen-presenting cells (APCs) [31–33]. These peptides are targeted to special vesicles where they form complexes with MHC II molecules. The MHC-peptide complexes are transported to the cell surface to bind T cells (as shown in Figure 2).

Each T-cell expresses a unique *αβ* TCR that specifically recognizes a particular MHC–antigen complex [34–36]. The ability of the T-cell repertoire to recognize a wide array of MHC-peptide combinations is attributed to the presence of a variety of germline V*α* and V*β* gene segments that can be shuffled and rearranged with the appropriate D*β*, J*β*, and J*α* genes to form the variable units of the TCR. These variable units are then combined with C*α* or C*β* gene segments to form a contiguous unit that encodes the mature *α* or *β* polypeptide chains. The junctional regions between V*β*:D*β*, D*β*:J*β*, and V*α*:J*α* create the hypervariable CDR3 domain of the TCR that recognizes MHC-peptide complexes with a high specificity [34]. In contrast to antigens, SAgs primarily interact with T cells through V*β* elements with low contribution from the other variable elements of the TCR. Each SAg has a signature specificity for a set of V*β* families and can interact with all T cells expressing those V*β* elements, regardless of the antigenic specificity of their TCR.

The roles of APC and MHC II molecules in presenting antigens and SAgs are quite distinct. In contrast to conventional antigens, SAgs do not require processing by APCs, and their activity is hindered by fragmentation as it disrupts the bridge between T cells and APCs [37]. After the binding of SAgs to the TCR and/or to MHC II molecules, intracellular biochemical signals that program several events leading to cell activation, differentiation, proliferation, and the release of inflammatory cytokines are triggered [38, 39]. Additionally, SAgs have a unique feature in that they can interact with many T cells that share particular sequences within the variable region of the *β* chain of the TCR, known as the V*β* element [40, 41]. SAgs have developed various mechanisms to optimize individual efficiency, resulting in their ability to attach to MHC II molecules in simple ways.

SAgs can interact with MHC II molecules outside the antigen groove, indicating that the binding of SAgs to MHC II is not restricted to the antigen groove [41, 42]. In addition, SAgs can bind to at least two distinct sites on MHC II molecules [43–45].

**Figure 3:**
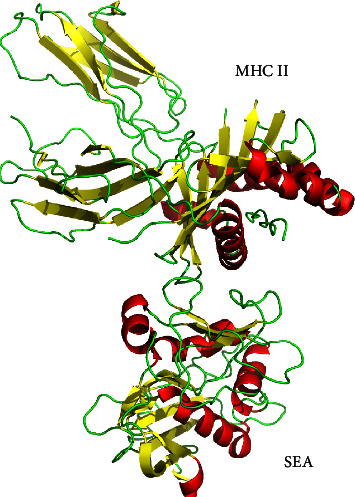
Crystal structure of SEA in complex with human MHC class II. Data from RCSB (https://www.rcsb.org, entry ID: 1LO5).

**Figure 4:**
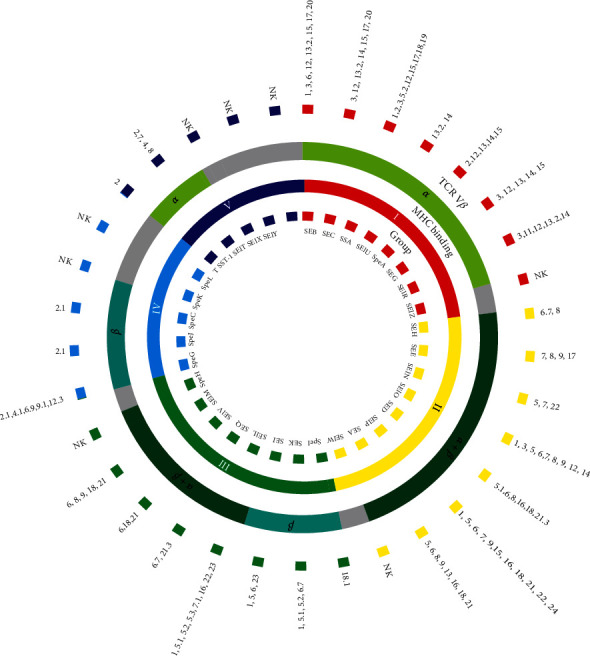
MHC Ⅱ binding sites and SAgs stimulating specific T cells with TCR V*β*.

Most SAgs use a common, overlapping, low-affinity generic binding site involving the *α*-chain of MHC II [46, 47], while some SAgs, such as SEA, use a second high-affinity, zinc-dependent binding site on the polymorphic *β*-chain (Figure 3) [43–45]. In general, SAgs bind to MHC II through either the invariant *α*-chain or the polymorphic *β*-chain (as summarized in Table 1, Figure 4) [9]. Cocrystallization studies of staphylococcal enterotoxin B (SEB) bound to the *α*-chain of MHC II have revealed a hydrophobic loop region exposed within the N-terminal *β*-barrel domain. This loop region binds to a hydrophobic groove located in the distal region of the DR *α*1-domain with binding affinities of 10^−5^ M [60, 61]. The polar binding pocket of SEB contains a glutamate and two tyrosine residues that accommodate Lys39 of the *α* subunit of MHC Ⅱ, while the hydrophobic region consists of a leucine and flanking residues that make several contacts with the MHC Ⅱ *α* chain. Interestingly, the MHC Ⅱ-binding sites of TSST-1 and SEB significantly overlap. The hydrophobic binding contacts of other SAgs with the MHC Ⅱ *α* chain have been proposed to be similar to those found in SEB and TSST-1 [45]. A conserved motif consisting of leucine in a reverse turn is conserved among bacterial SAgs [45, 60], which may provide the key determinant for binding MHC Ⅱ either by the hydrophobic pathway or otherwise.

However, TSST-1 does not have a highly charged residue in the polar pocket that interacts with Lys39 of the MHC Ⅱ *α* chain. Instead, TSST-1 uses an alternative conformational binding mode that allows it to interact with MHC Ⅱ *β*-chain residues and the carboxy-terminal region of the antigenic peptide. Some SAgs, such as SEA and SEE, bind to the *β*-chain of the MHC II molecule at a high-affinity site, which is distinct from the generic binding site [61]. This high-affinity site is formed by the amino-terminal domain of SEA interacting with the MHC II *α* chain and by the carboxy-terminal domain residues of SEA (His187, His22S, and Asp227) forming a zinc-coordination complex with His81 from the *β* chain of an adjoining MHC II molecule [62]. This site has a 100-fold greater affinity than the generic binding site and is used by SEK, SEI, and group IV SAgs [54, 63, 64]. Table 1 provides further details on the specific SAgs that utilize this high-affinity binding site, including SEK, SEI, and group IV SAgs.

SPE-C is the first streptococcal SAg that has been shown to exhibit a high-binding mode. Its binding to the MHC II *β*-chain can be completely abolished by adding EDTA and restored by excess Zn2^+^ over EDTA [65]. The cocrystal structure of SPE-C with the MHC II molecule also revealed extensive interaction with the bound peptide [66]. Structural analysis of SPE-H, SPE-J, and SMEZ-2, and computer-generated models of SPE-G, SPE-I, SPE-K, SPE-L, and SPE-M showed that they also have a conserved zinc-binding site in their C-terminal domain, but lack a generic MHC II *α*-chain binding region. Additionally, Groups II and III SAgs have an *α*- and *β*-chain binding region but do not directly stimulate T cells upon binding to MHC II molecules [45]. However, they increase the potential of bound SAgs to interact with the *α* chain of another MHC Ⅱ, thereby increasing their biological potency.

### 3.2. T-Cell-Activation and Proliferation

Antigen-driven activation and differentiation of T and B cells is a complex process that requires specific cognate interaction between the TCR/CD3 complex and antigen presented in the context of MHC II molecules on the surface of B cells [67]. This interaction is MHC allele restricted and dependent on the availability of V_H_ elements capable of recognizing the antigen MHC II complex. However, when a toxin binds to MHC II, it behaves as a superantigen that interacts with T cells via the TCR V*β* chain, inducing MHC-unrestricted T-cell activation and proliferation [2, 68]. TCR binding is specific to the variable region V*β* of the receptor [69, 70], and even small amounts of SAgs presented on dendritic cells are sufficient to initiate the T-cell response [71]. This results in massive V*β*-dependent T-cell proliferation and subsequent release of proinflammatory cytokines [9, 12], which cause a cytokine storm known as TSS [72]. Upon binding of the endogenous antigen to MHC II, the APC releases IL-1 and recruits CD4^+^ T cells to bind to the antigen, which subsequently stimulates B cells to differentiate into memory and plasma cells. In conjunction with antigen binding to MHC II, CD4+ T cells release IL-2, which stimulates CD8+ T cells to differentiate into Tk cells or memory T cells. In addition, exogenous antigens are processed by APCs and directly presented to CD8+ T cells, stimulating the proliferation and differentiation of CD8+ T cells. Humoral and cellular immunity work together to eliminate pathogenic microorganisms invading the body. Therefore, SAgs play a similar role in immune activation, as studies have shown that CD4+ T cells mediate the proliferation and differentiation of B cells stimulated by SAgs [73] and activate CD8+ T cells [74].

It is noteworthy that despite the ability of SAgs to bind to the lateral surface of MHC II molecules [42], thereby circumventing their antigen-specific peptide binding region, T-cell activation by SAgs remains partially selective. Different SAgs are known to stimulate specific T cells with TCR V*β* to proliferate, as shown in Table 1, which can result in TCR repertoire skewing [75]. This phenomenon can have a severe effect on the host, including T-cell clonal deletion in mature T cells [2]. In developing T cells, this may result in apoptosis and negative selection of the TCR repertoire [76]. Consequently, the immune dysregulation caused by SAgs can result in serious autoimmune or immune-tolerant diseases in the host.

### 3.3. Mucosa-Associated Invariant T (MAIT) Cells Activation

The innate immune system is the body's first line of defense against bacterial infections [77]. This system has specialized receptors known as pattern-recognition receptors that can detect molecular components of invading pathogens, called pathogen-associated molecular patterns, and act as an early surveillance system [78, 79]. Once innate immune receptors detect foreign molecular patterns that are distinct from those found in host cells, innate immune receptors quickly elaborate antimicrobial effector molecules, such as cytokines that act as an initial defense and influence the ensuing adaptive immune response.

In contrast, adaptive immunity takes longer to develop, but it is often necessary to fully eradicate the pathogen [80]. However, innate T cells are capable of acting during this critical lag time while adaptive immune responses develop. Innate T cells also possess somatically rearranged TCR that have undergone thymic selection. Unlike their conventional counterparts, innate T cells recognize nonpeptide microbial molecular patterns (or danger signals) and rapidly produce effector functions after activation [81]. MAIT cells are a subset of such unconventional T cells that have gained attention. These cells exist in expanded numbers in adult humans and have a mature phenotype ready to respond to antigens. The differentiation and maturation of MAIT cells can be observed in fetal tissues [82], while the expansion to 1%–10% of T cells in peripheral blood occurs after birth [83].

MAIT cells are characterized by conserved and invariant TCR features, which enable them to recognize antigens in complex with MHC-Ib-related protein 1 (MR1) [84]. Indeed, studies have shown that MAIT cell activation is blocked by anti-MR1 antibodies [85, 86]. Once activated, MAIT cells have been found to possess innate antimicrobial activity [87, 88]. Notably, they are activated only by microbes that have the riboflavin biosynthetic pathway (such as Streptococcus Group A) [85, 86]. This suggests that the riboflavin biosynthetic pathway plays an important role in MAIT cell activation, although other pathways of activation may also exist. Interestingly, several in vivo pathogen infections were shown to activate invariant natural killer cells via IL-12 rather than through recognition of cognate antigens [50, 87, 89]. Thus, during acute viral or microbial infection, MAIT cells recruited to the site of infection may act as amplifiers of the innate antiviral response. While strong activation of an immune cell subset such as MAIT cells can be beneficial, it can also pose a severe threat to the host. MAIT cells represent up to 10% of T cells in the blood and up to 35% in the liver and some mucosal sites [83, 84, 90, 91], and an excessive immune response may cause severe inflammatory disease.

Some reports suggest that SAgs, such as SEB, activate MAIT cells and induce substantial production of proinflammatory cytokines. This response relies on MHC II molecules and IL-12 but not MR1. In addition, inhibitors of components of the mitogen-activated protein kinase pathway were able to block this MAIT cell response. This activation mode indicates that specialized APCs, such as dendritic cells, can initiate this mode of MAIT cell activation. Interestingly, *S. aureus* has the riboflavin biosynthesis pathway and generates MR1-presented antigens that MAIT cells can detect [86]. Thus, when *S. aureus* primarily invades the host, the antigens and SAgs can activate MAIT cells simultaneously, posing a threat to the survival of the host and itself simultaneously. The hyperactivation of MAIT cells induced by SAgs can result in an unresponsive state, known as “anergy,” upon subsequent encounters with MR1-presented bacterial antigens. Unlike other T cells, MAIT cells do not produce memory cells upon antigen stimulation, resulting in persistent anergy. However, while only a small fraction of MAIT cells expressing the TCRV-*β* fragment are activated by SEB, the strong MAIT cell activation and IFN*γ* production are primarily driven by IL-12 and IL-18 produced by other cells in peripheral blood mononuclear cells following SEB-mediated activation of polyclonal T cells.

In this system, the anergic state is associated with the induction of inhibitory receptors such as T-cell immunoglobulin and mucin-3 and lymphocyte-activation gene 3 (LAG-3) on MAIT cells, and blocking LAG-3 could restore their responsiveness to bacteria. In addition, T-cell anergy should not be dismissed since SAgs can activate not only MAIT cells but also polyclonal T cells, which can generate memory cells when stimulated by antigens. Overall, both T-cell anergy and SAgs contribute to the shielding of memory cell function, which allows *S. aureus* to colonize the host.

### 3.4. T-Cell Anergy and Deletion

The specific ability of SAgs to interact with the variable *β* domain of the TCR has provided an opportunity to examine the fate of reactive T cells in vivo, independent of functional assays. Such studies have revealed that activated T cells can undergo proliferation, anergy, or apoptosis [92, 93]. In vivo recognition of endogenous SAgs leads to the intrathymic deletion of V*β*-specific subsets during the double-positive (CD4+, CD8+) stage of development. This deletion subsequently manifests in both mature CD4+ and CD8+ subsets [94–96]. In the case of exogenous SAgs, an early report showed that newborn mice injected with SEB virtually lacked mature thymocytes expressing V*β*3+ and V*β*8+. This finding demonstrated for the first time that clonal deletion can accompany induced immunotolerance to a foreign antigen [2]. Further research has confirmed that SEB-specific mature T cells undergo an initial expansion followed by anergy induction in both in vivo and in vitro models [51, 97]. Furthermore, SEB-induced death of V*β*8+ cells occurs independently of the intact thymus and is also observed in adult animals that have undergone thymectomy [98].

### 3.5. SAgs as Mediators of Human Diseases

One of the most fascinating aspects of SAgs is their potential involvement in various human diseases, as summarized in Table 2. The pathogenesis of SAg-associated diseases is suggested to be mediated by aberrant immune responses elicited in response to these molecules. The ability of SAgs to interact with a vast number of T cells and induce the production of high levels of inflammatory lymphokines and monokines makes them active in the pathogenesis of food poisoning, TSS, sudden infant death syndrome, and several autoimmune diseases. However, it is important to distinguish between diseases in which a direct link with a particular superantigen has been established and those in which the role of SAgs is highly suspected but not yet confirmed.

Pathogenic microorganisms often secrete a variety of SAgs after infecting the host, which can trigger a strong immune response and severe inflammatory reactions, potentially leading to an early host. However, scientists have found that SAgs may play a role in promoting the evolution of pathogenic microorganisms.

By inducing a strong nonspecific immune response, SAgs may help these microorganisms achieve an equilibrium state with the host and eventually coexist with it. Rather, SAgs induce T-cell anergy, causing the immune system to lose its ability to respond to pathogenic microorganisms. They further contribute to the colonization of pathogenic microorganisms in vivo, leading to the development of many diseases.

The mechanism of SAgs-induced diseases is quite complex, as pathogenic microorganisms usually release multiple types of SAgs. Despite the complexity, the host's TCR repertoire skewing demonstrates that only one or a few specific TCR V*β* are activated.

Furthermore, T-cell anergy by SAgs helps protect the host from SAg-induced damage, such as when T cells that have completed their function are eliminated or as a result of disease or medical treatment. Nevertheless, excessive T-cell anergy can lead to immunotolerance and increased susceptibility to infection. This can lead to a range of diseases, such as systemic lupus erythematosus, rheumatoid arthritis, and multiple sclerosis (MS). In the following section, we will discuss selected examples of acute and chronic diseases that have been associated with SAgs. The most dangerous examples are infectious diseases caused by SAgs, such as TSS, in which a severely infected person loses vital signs for a short time. In addition, SAgs are responsible for cell transformation and carcinogenesis, and immune activation of SAgs may be involved in tumorigenesis.

### 3.6. Infectious Diseases

Bacterial, viral, and other microbial infections can result in the production of SAgs and cause the body's immune response, thus leading to infectious diseases, such as TSS, food poisoning, severe acute respiratory syndrome (SARS) [106], and sepsis. A common characteristic of infectious diseases is the ability of SAgs to induce hyperactivation of the immune system, leading to multiple organ failure and potentially fatal outcomes. TSS is a rare but potentially life-threatening bacterial infection caused by certain strains of *S. aureus* bacteria that produce SAgs. These SAgs cause a massive immune response in the body, leading to symptoms such as fever, rash, low blood pressure, and, in severe cases, organ failure. TSS can occur in anyone but is more common in women who use high-absorbency tampons [108]. The main culprits that cause TSS are Group A streptococci and *S. aureus*. While Group A streptococci is an aerotolerant anaerobe, *S. aureus* is a facultative aerobe. Tampons introduce oxygen into the anaerobic vagina [109, 110], which can encourage the growth of *S. aureus* and the production of SAgs.

These SAgs trigger a cytokine storm, causing the release of tumor necrosis factor-alpha (TNF-*α*), tumor necrosis factor beta (TNF*β*), interleukin (IL)-1, and IL-2 from activated T cells and APCs [40, 111]. TNF-*α* is the primary mediator of shock, and anti-TNF-*α* has been shown to inhibit the progression of SAg-driven shock in mice [112]. The rapid progression from onset to multiorgan system failure in TSS necessitates immediate action with aggressive fluid resuscitation and concomitant respiratory and often inotropic support. The next subsection will discuss strategies for blocking and treating SAgs-induced TSS.

### 3.7. Autoimmune Diseases

SAgs have been identified as potential threats, playing a role in the development of several autoimmune diseases. The activation of T cells by SAgs results in the secretion of a plethora of cytokines, which can lead to tissue damage and chronic inflammation. Some examples of autoimmune diseases associated with SAgs include rheumatoid arthritis [113–115], systemic lupus erythematosus [116], and MS [104, 117]. In these diseases, it is believed that the presence of SAgs may trigger an inappropriate immune response against self-antigens, leading to autoimmune pathology.

Infections that occur naturally in animals are characterized by demyelination, which can be seen in diseases caused by viruses such as Theiler's murine, encephalomyelitis virus, canine distemper virus, MHV, Visna virus, and caprine arthritis-encephalitis virus [118]. Microbial infection can be used as a starting factor to induce autoimmunity and lead to clinical manifestations in genetically susceptible individuals. Some studies suggest that MS, similar to other autoimmune diseases, may be triggered by microbial infections [119]. EBV, a double-stranded DNA herpes virus, is one such infection that may increase the risk of MS [120].

Human herpes virus 6 (HHV-6) is another double-stranded DNA virus that can cause fever, encephalitis, and other diseases. Research has suggested that HHV-6 may have a pathogenic effect in MS, as the HHV-6 antigen has been detected in the cerebrospinal fluid of some MS patients [121]. In addition, viral antigens were detected in neurons, microglia, and lymphocytes of MS patients [122]. While neither EBV nor HHV-6 carry genes in their own genomes that encode SAgs, studies have shown that their infections cause an inflammatory response in the central neuronal system (CNS) in which HERVs are activated. HERV is a type of human DNA that makes up approximately 8% of the human genome. Under normal physiological conditions, epigenetic modification silences HERV gene expression, whereas under certain pathological conditions, EBVs can deactivate HERV-K21 Env protein expression in resting B lymphocytes through CD18 receptor interaction [22, 123, 124]. This deactivation produces viral transcripts and encodes proteins with superantigen properties, causing a severe immune response leading to oligodendrocyte death. HERV may be involved in many diseases, especially autoimmune diseases and neoplastic diseases [125]. The isolation of HERV from the cerebrospinal fluid of MS patients suggests that HERV may play a pathogenic role in MS [126, 127]. It also leads to neuroinflammation and oligodendrocyte apoptosis [127].

Afterward, researchers discovered that HERV-W Env-encoded synsporin-1 was upregulated in astrocytes and microglia in MS patients and was associated with active demyelination [128]. Autoreactive T cells are activated during peripheral infection by infectious agents and can penetrate the blood–brain barrier into the CNS to cause an inflammatory response. This can cause infectious agents to present amino acid sequences such as host autoantigens or when the antigens of infectious agents spread through epitopes, inducing the immune system to attack the tissues, resulting in tissue damage [129, 130]. When T cells activated by SAgs encounter the antigen presented by APCs, especially microglial cells, they initiate the local inflammatory process in the CNS, causing severe inflammation of the CNS [131, 132] and leading to severe neurological dysfunction [133]. While increased HERV expression in MS patients is not sufficient to prove a correlation between HERV and MS etiology, subsequent studies have shown that SEB exacerbates clinical symptoms in human MS patients [134]. This suggests that EBV or HHV-6 activation of HERV expression contributes to MS progression to some extent.

### 3.8. Mantle Cell Lymphoma (MCL)

The development of B cells and T cells involves the introduction and repair of DNA double-strand breaks to create functional receptors [135]. In this process, faulty DNA recombination can lead to the overexpression of proto-oncogenes, resulting in the uncontrolled proliferation of individual lymphocytes and, eventually, transformation into lymphoma [136]. In fact, approximately 90% of MCL cases arise from mutations in B cells [137]. MCL is a subtype of non-Hodgkin's lymphoma characterized by the abnormal growth and accumulation of B lymphocytes in the mantle zone of the lymphoid follicles [136]. Studies have shown that the BCR signaling pathway plays an important role in the pathogenesis and progression of MCL, with activated BCR signaling found in B cells in lymphoma [138–141]. In particular, *S. aureus* protein A (SpA) has been identified as a potent activator of T cells or B cells and is believed to be involved in the development of MCL [142]. SpA is known to bind to specific motifs composed of 13 amino acids in the TCR and BCR framework regions [143, 144]. While these motifs are often mutated in normal individuals, the low mutation rate of the IGHV3 family in MCL cells means that SpA may activate BCR and promote the proliferation of B cells. Studies have shown that after SpA exposure, the number of B cells expressing the IGHV3 gene is reduced, possibly due to the overactivation of B cells caused by SAg and apoptosis.

However, early lymphoma B cells may overcome this lack of signaling with the activation of B cells, allowing mutated B cells to escape apoptosis and eventually transform into tumors [143]. The IGHV3 family is the most abundant IGHV family, and approximately half of MCL cells express IGHV3 genes. Studies have shown that these BCRs can also be activated by SpA [142]. However, in normal individuals, the SpA-binding motifs are often mutated, which means that SpA may not activate BCR. On the other hand, the IGHV3 family in MCL cells has a low mutation rate. Studies have shown that after SpA exposure, the number of B cells expressing the IGHV3 gene is strongly reduced, which may be due to the overactivation of B cells caused by SAg and apoptosis [145]. However, early lymphoma B cells may overcome this lack of signaling with the activation of B cells. Because entire B-cell subsets are activated or proliferated, some B cells that have mutated may escape apoptosis and eventually transform into tumors. SpA further stimulates the proliferation of lymphoma B cells as MCL cell BCRs retain their proliferative function [136]. Thus, early SAg-activated substantial B-cell activation may be the first step in the development of lymphoma, and SAgs further promote the progression of MCL.

## 4. Therapeutic Interventions

The impact of SAgs on various diseases highlights the importance of choosing an appropriate therapeutic intervention based on the severity and specific type of disease, as well as the patient's overall health and medical history. Fortunately, there are multiple therapeutic interventions available for treating SAgs-induced diseases.

### 4.1. Antimicrobial Treatment to Reduce the Production of SAgs

Patients with severe TSS face a higher mortality rate when initial antibiotic therapy is inadequate. However, there is a scarcity of clinical trial data on antibiotic regimens for TSS. In vitro studies and theoretical considerations suggest avoiding the use of certain antibiotics, such as *β*-lactam drugs and lincoamide. Instead, it is recommended to wait for culture results before starting treatment. The main goal of the treatment should focus on reducing exotoxin production and microbial load. In cases when the causative organism is unknown, broad-spectrum antibiotics or a combination of antibiotics may be used for treatment. However, antimicrobial treatment could lead to the creation of super bacteria that render antibiotics useless.

### 4.2. Intravenous Immunoglobulin (IVIG)

IVIG is commonly used to treat TSS caused by staphylococcal and streptococcal SAgs. When SAgs bind to TCR and MHC II molecules, the cytokine expression of T cells (mainly lymphotoxin *α*, IL-2, and interferon-*γ*) and APCs such as monocytes (mainly TNF, IL-1*β*, and IL-6) rapidly increases. This increase may be due to the activation of the transcription factor nuclear factor *κ*B (NF*κ*B) [146], which plays a central role in the generation and extension of inflammatory responses, coagulation activation, and development of organ dysfunction. The degree of NF*κ*B activation has also been associated with a high risk of death [147, 148]. Indeed, T-cell activation leads to the recruitment of more T and B cells to the site of infection, clonal T-cell expansion, and activation of APCs, which further amplify the release of proinflammatory mediators and increase procoagulant activity. The released cytokines, including interferon-*γ*, rapidly induce the occurrence of TNF and IL-6, resulting in a complex interplay and a cytokine storm, which leads to toxic shock [149].

Moreover, patients with TSS antibody deficiency are at a higher risk of developing and recurring TSS. Previous studies showed that the concentration of SAgs neutralizing antibodies was significantly reduced in patients with invasive Group A Streptococcal infection [150, 151]. However, treatment with IVIG has been proven to improve outcomes in patients with TSS [152–154]. IVIG is a blood-derived product made from thousands of healthy blood samples. It contains mainly monomeric, purified, multispecific immunoglobulin G and smaller fractions containing other immunoglobulin isotypes and immune components [155]. An injection dose of 2 g/kg IVIG has good anti-inflammatory and immunomodulatory effects in the treatment of TSS, mainly including promoting antigen recognition and activating innate immune recognition. Additionally, IVIG can activate the innate immune system and block the activity of many SAgs by neutralizing antibodies [156–158]. This can effectively block SAgs-induced T-cell activation [159, 160]. Additionally, it is important to note that the use of IVIG as an adjunctive therapy in patients with TSS can be limited by the acute nature of the disease. If the SAgs level in the patient's blood declines rapidly, there is a risk of delayed treatment, leading to worse outcomes. Therefore, IVIG is most effective only when given in the very early stage of TSS [161].

### 4.3. Peptide Antagonists and Receptor Mimetics

To activate the immune system, SAgs need to bind to both of the major histocompatibility complexes, MHC II and TCR. Any disruption to this mechanism during this period will prevent SAgs from activating T cells. A previous study screened various short peptides from the SEB domain and identified a dodecapeptide that weakly antagonized SEB activity. This peptide was found to be distant from the known binding sites of MHC II and TCRs, and it inhibits SAg-induced human IL-1, interferon-*γ*, and TNF-B gene expression [162]. The specific function of this peptide is unknown, but it is a conserved region of SAgs and has a broad spectrum of SAgs antagonist properties [163, 164]. It is worth to mentioning that this peptide does not participate in the binding of MHC II to TCR and does not inhibit the activation of T lymphocytes in vitro.

Based on how SAgs activate the immune system and counteract their effects, researchers have designed bispecific receptor mimics that target MHC II binding by linking to the TCRV*β* binding site of SAgs with a peptide linker [165]. Depending on the TCRV*β*-binding site receptor, these specific mimics can block different SAgs [166]. It is important to note that specific receptor mimics and MHC Ⅱ or TCR all compete for binding SAgs. Therefore, the affinity of specific receptor mimics to SAgs can be enhanced through site-directed mutagenesis or specific modification [167]. There can be one SAg that can bind to multiple TCRV*β* receptors, so it is often necessary to design multiple TCRV*β* receptors according to one SAg. Overall, these results suggest that specific receptor mimics could be a promising strategy for blocking SAgs and modulating immune responses.

With a better understanding of SAgs, researchers have found that full activation of T cells requires both B7-2 and B7-1 costimulatory receptors to bind CD28 [168]. This activation leads to the release of inflammatory cytokines [169–171]. CD28 is a homodimer expressed on T cells that acts as a major costimulatory ligand in immune responses by interacting with the B7 receptor. While B7-2 is constitutively expressed, B7-1 is only induced during the immune response to CD28 signaling [172, 173]. As such, the B7-2–CD28 interaction is responsible for regulating the inflammatory response, while the weak interaction between CD28 and B7-2 ensures moderate signaling of the inflammatory response to promote immune protection while avoiding cytokine storms [168, 174]. CD28-regulated signal transduction pathways are activated in T-cell SAgs-stimulated species, which affects SAg-induced IL-2 gene expression through the CD28 response element contained in the IL-2 gene promoter [175]. Other studies conducted by Saha et al. [176] have shown that CD28-deficient mice are completely resistant to lethal toxic shock induced by TSST-1, suggesting that preventing SAgs from binding to CD28 is sufficient to prevent SAgs-induced lethality. Therefore, researchers have identified the CD28 homodimer as the key receptor target of SAgs to design CD28 mimetic peptides to selectively and competitively bind to CD28 with SAgs activity being blocked [176]. For instance, the CD28 antagonism simulation peptide AB03 has been shown to protect mice from deadly poisons caused by streptococcus outside the body. Similarly, the CD28 antibody E18 effectively blocked the binding of CD28 to B7 molecules and inhibited the activity of T cells in graft-versus-host disease. However, T-cell activity was further suppressed after the injection of CD28 antibody E18 in healthy mice, suggesting that CD28 peptide E18 simulations can be used as CD28 signal transduction inhibitors [177]. In addition to the above methods, there are several other drugs available that have shown efficacy in blocking SAgs, such as S101 and gamma globulin [178, 179]. These drugs work by inhibiting the proliferation of T cells, which in turn helps rescue the host from SAg-induced threats. Receptor mimetic peptides are an excellent therapeutic strategy for SAgs-caused diseases and are highly likely to be used in the treatment of graft-versus-host disease [177]. However, excessive immunosuppression can weaken the immune system, leading to other infections or diseases.

## 5. Nanorobots for Blocking SAgs

Although current treatment strategies for blocking SAgs have limitations, combining different approaches may help to overcome their shortcomings. However, effective drug delivery is crucial for successful treatment. In recent years, bionanorobot intelligent drug delivery systems have received significant attention in biomedical applications due to their highly customizable nature. One promising approach is using DNA origami of single-stranded scaffolds to create custom shapes through the interactions of hundreds of oligonucleotide strands., which allows for the precise arrangement of different components [180]. The biocompatibility of DNA nanorobots avoids toxicity, immunity, and other biological side effects while also enabling penetration into in vivo barriers such as the epithelium, endothelium, and cell membrane. Moreover, nanorobots can be loaded with multiple drugs for the combined treatment of a single disease. Intelligent system delivery of DNA nanorobots can help to avoid the side effects of drugs by incorporating intelligent targeting and drug release mechanisms.

To improve the accuracy of drug delivery, various intelligent materials have been proposed that can also respond to pH, temperature, magnetic fields, and other stimuli. Currently, state-of-the-art drug delivery systems with active targeting are based on monoclonal antibodies as guide ligands. Indeed, the given specificity of antibody–antigen interactions allows precise labeling of diseased cells that overexpress a certain receptor. Often, when a disease cannot be characterized by a single specific marker, more complex drug-delivery systems are needed.

Active targeting in drug delivery systems has progressed significantly with the use of monoclonal antibodies as guide ligands. Antibody–antigen interactions exhibit high specificity, enabling precise labeling of diseased cells that overexpress a particular receptor. However, when a disease lacks a single specific marker, more complex drug-delivery systems are needed.

**Figure 5:**
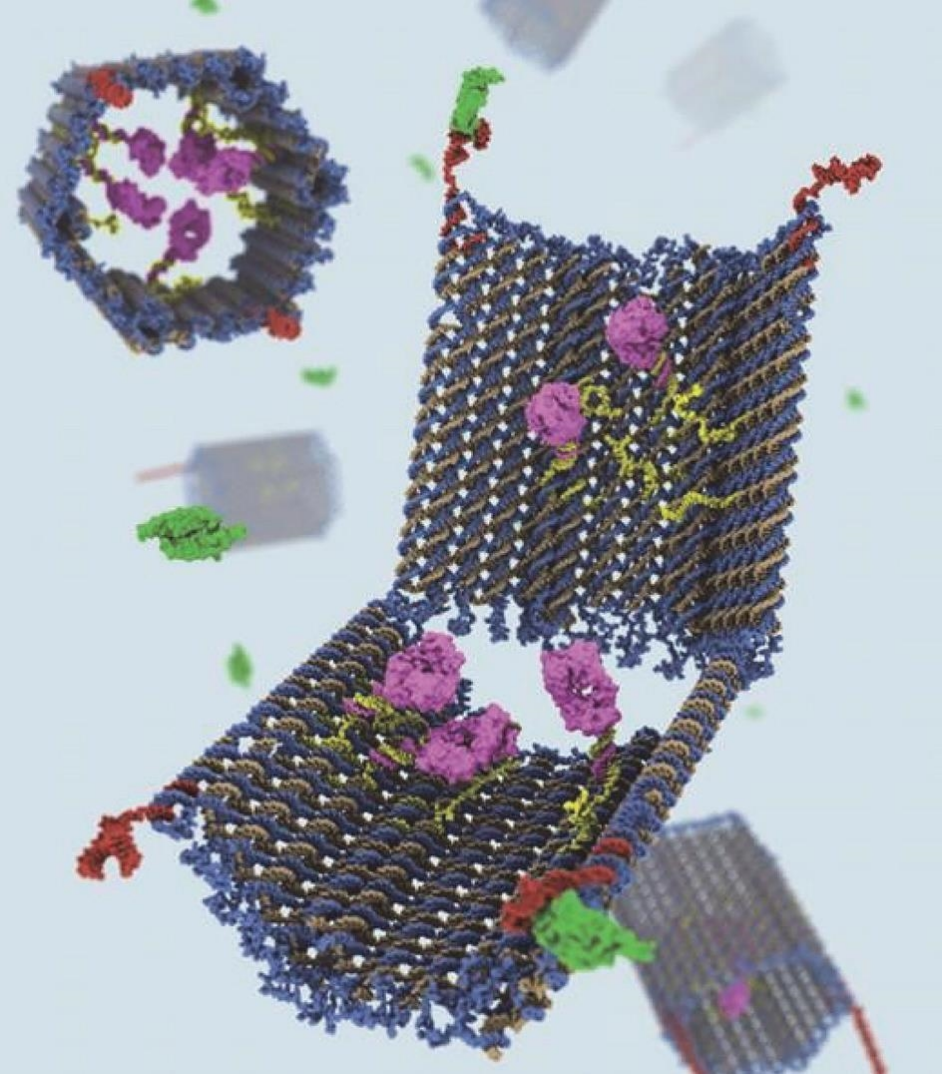
A DNA nanorobot springs open like a clamshell to reveal its payload, antibody drugs (purple). The DNA shell is held together by 2 DNA locks (red) that open when they meet molecular keys (green) found on the surface of a cell. The top left shows a side view of the DNA shell in its closed state.

In such cases, Boolean logic is frequently used to specify the presence or absence of biomarkers as Boolean true and false values, respectively. Therefore, processing the input data computationally using biomolecular logic circuits distinguishes diseased cells and tissues from their healthy counterparts, significantly improving therapeutic efficacy and diagnostic accuracy. Researchers have extended the work by designing a box using DNA origami that releases its drug load only in the presence of a specific target molecular configuration linked by switchable hinges (Figure 5) [181].

The box is equipped with switchable hinges, and two different DNA aptamers are used to turn off and lock the DNA nanorobots. Each aptamer is designed to specifically bind to different protein antigens, allowing the nanorobots to sense signals on the cell surface for conditional triggering activation and form an AND logic gate, requiring the activation of two protein targets for drug release.

When the two aptamers successfully bind their respective targets, the DNA nanorobot changes its conformation and releases the drug-loaded within. Thus, the aptamer-encoded locks act as a sensory-computation-driven mechanism that can be deployed to trigger specific therapeutic responses. Furthermore, nanorobots have several advantages, including the ability to use fluorescent markers, track biomarkers, collect information about the surrounding environment, and visualize essential molecular pathways and functions in cells, tissues, and organs.

This allows for early diagnosis at the molecular and cellular levels before tissue damage occurs or typical symptoms of disease appear. Fluorescent-labeled nanorobots can provide precise information about the location and function of drugs in real-time within the intercellular and intracellular space. This endows nanorobots with high spatiotemporal traceability, sensitivity in complex biological environments, fluorescence mobility, and external navigation, all of which make them useful in targeting complex biological environments.

Research has demonstrated that nanorobots loaded with antibody fragments can effectively respond to two distinct types of cell signaling stimuli [180]. To leverage this capability, molecular switches were designed based on the *α* and *β* binding sites of SAgs and MHC Ⅱ molecules, respectively. These switches were integrated into DNA nanorobots, which were then loaded with peptide antagonists, IVIG, or mimetic receptors such as CD28 mimetic peptides. Upon encountering SAgs, the molecular switch triggers a change in the nanorobot's structure, leading to the release of the loaded antibodies. This targeted delivery approach aims to reduce the impact of these drugs on T-cell activity in healthy hosts, achieving precise and effective treatment outcomes.

In addition, SAg is also being explored as a potential immunotherapeutic agent for cancer treatment. They have been shown to induce the activation and proliferation of T cells, which recognize and kill cancer cells. A previous study has shown that mutating inactivates emetic activity sites of SEC2, such as Cys93, Cys110, and His118, which can inhibit tumor growth while eliminating SEC2 as a therapeutic side effect [182]. However, because SAgs also contribute to tumor progression to some extent, the use of SAgs in cancer treatment is still experimental, and more research is needed to fully understand their potential benefits and risks.

## 6. Conclusion

SAgs are proteins encoded mainly in bacteria and viruses that cause the activation of a large number of T cells. To date, 26 staphylococcal SAgs and 11 streptococcal SAgs have been identified, as well as viral-encoded SAgs and HERV-encoded SAgs due to inflammatory environments. SAgs have been divided into five groups based on their amino acid sequences and structural features. This classification has guiding significance for the development of SAgs blocking drugs and disease treatment. Unlike antigens, SAgs do not need to be processed by APCs but directly bind to the MHC II antigen-binding domain and the side of the TCR in the form of intact proteins, thereby activating T cells. This implies that T-cell activation by SAgs is not MHC-restricted and becomes the basis for the activation of a large number of T cells. However, T-cell activation of SAgs is restricted to specific TCR V*β* regions, and different SAgs can activate different TCR V*β* cells (Figure 2). SAgs can lead to the elimination of specific types of T cells during immune system development. However, in mature individuals, SAgs can lead to the functional impairment of some T cells, making the host significantly susceptible to some pathogenic microorganisms.

Excessive activation of T cells can cause the release of a large number of immune factors, leading to severe cytokine storms and toxic shock in the host. In addition, the impairment of T-cell function caused by SAgs can lead to defects in the host immune system, which can trigger autoimmune diseases such as MS. In addition, excessive activation of B cells may also lead to abnormal B-cell proliferation, which in turn can form cancer cells. Although the mechanisms by which SAgs activate the immune system are well understood, blocking SAgs remains a major challenge. One of the main reasons is that SAgs, as metabolites of microorganisms, have a very complex composition. In addition, microorganisms often release multiple SAgs to activate different TCR V*β* T cells and determining which SAgs function or cause disease etiology is very difficult, making SAgs and antibody development difficult. For toxic shock, timely treatment is essential. Therefore, the injection of immunoglobulin to neutralize SAgs is a very effective first-aid method. For autoimmune diseases caused by SAgs, long-term exposure to SAgs can cause damage to host tissues; therefore, blocking SAgs can slow the damage of the immune system to host tissues and disease progression. Advances in mimetic peptide technology have made it possible to develop antibodies that block SAgs. The mimetic peptide acts as a competitive binding molecule of CD28 and attenuates the activity of superantigen-induced T cells by competitively binding the CD28 binding site. Although these peptides do not act as costimulators to activate T cells, they may also prevent antigen binding to T cells, thereby increasing the risk of infection. Although mimetic peptides offer a promising means of blocking SAgs, it is important to ensure that they do not interfere with normal host immunity. Therefore, controlling the release of the mimic peptide/drug at the right time is essential for its effectiveness.

In recent years, advances in drug delivery technology have led to the emergence of smart nanorobots, a novel drug delivery system, to enable precision medicine. The system consists of three parts: an aptamer switch targeting the tissue/antigen, a carrier to deliver the antibody/drug, and the antibody/drug itself. Once injected into an organism, aptamers actively seek out and recognize the target tissue and trigger antibody/drug release from the vector. This mechanism ensures precise blocking and prevents any possible adverse side effects due to imprecise drug targeting. Therefore, intelligent bionanorobots are considered ideal delivery systems for blocking SAgs.

## Figures and Tables

**Table 1:** MHC Ⅱ binding sites and SAgs stimulating specific T cells with TCR V*β*.

SAgs	Group (new)	MHC binding site	Human TCR V*β*	References
SEB	I	*α*	V*β*1, 3, 6, 12, 13.2, 15, 17, 20	[2, 48–53]
SEC	I	*α*	V*β*3, 12, 13.2, 14, 15, 17, 20	[48, 49, 52, 53]
SSA	I	*α*	V*β*1,2,3,5.2,12,15,17,18,19	[53–56]
SElU	I	*α*	V*β*13.2/14	[9, 56]
SElZ	I	NK	NK	—
SpeA	I	*α*	V*β*2,12,13,14,15	[9, 52, 53, 56]
SEG	I	*α*	V*β*3, 12, 13, 14, 15	[48, 49, 55]
SElR	I	*α*	V*β*3/11/12/13.2/14	[9, 55, 56]
SEH	II	*α* + *β*	V*β*6.7,8	[48, 55, 56]
SElW	II	NK	NK	—
SElN	II	*α* + *β*	V*β*7, 8, 9, 17	[9, 49, 55]
SElO	II	*α* + *β*	V*β*5, 7, 22	[9, 49, 55]
SED	II	*α* + *β*	V*β*1, 3, 5, 6,7, 8, 9, 12, 14	[48, 49, 53, 55]
SElP	II	*α* + *β*	V*β*5.1/6/8/16/18/21.3	[9, 55, 56]
SEA	II	*α* + *β*	V*β*1, 5, 6, 7, 9,15, 16, 18, 21, 22, 24	[48, 49, 52, 53, 55]
SEE	II	*α* + *β*	V*β*5, 6, 8, 9, 13, 16, 18, 21	[48, 49, 52, 53]
SpeH	III	NK	V*β*9.1,12.6,23.1	[56]
SpeI	III	*β*	V*β*18.1	[56–58]
SElL	III	*α* + *β*	V*β*1, 5.1, 5.2, 5.3, 7.1, 16, 22, 23	[9, 55, 56]
SEQ	III	*α* + *β*	V*β*6.7, 21.3	[9, 55, 56]
SEK	III	*β*	V*β*1, 5.1, 5.2, 6.7	[9, 55, 56]
SEI	III	*β*	V*β*1, 5, 6, 23	[48, 49, 55]
SElV	III	*α* + *β*	V*β*6/18/21	[9, 55, 56]
SElM	III	*α* + *β*	V*β*6, 8, 9, 18, 21	[9, 49, 55]
SpeK	IV	*β*	NK	[56]
SpeL	IV	*β*	NK	[56]
SpeG	IV	*β*	V*β*2.1,4.1,6.9,9.1,12.3	[56, 59]
SpeJ	IV	*β*	V*β*2.1	[56]
SpeC	IV	*β*	V*β*2.1	[52, 53, 56]
SElX	V	NK	V*β*1/6/8/21	[9, 56]
TSST-1	V	*α*	V*β*2	[9, 49, 52, 53]
SElY	V	NK	NK	[9]
SElT	V	*α*	NK	[9]
SmeZ	V	NK	V*β*2,7, 4, 8	[56]

**Table 2:** Known and suspected association of SAgs with human diseases.

Disease	SAgs	References
Acute glomerulonephritis	SPE	[12]
Atopic dermatitis	Any	[99]
Bacteremia	SEIW	[100]
Desquamating syndrome in AIDS	TSST-1/SEB/SEC	[101]
Diabetes mellitus	HERV-K18	[28]
Food poisoning	SEs	[102]
Guttate psoriasis	SPE	[12]
Kawasaki syndrome	TSST-1	[75]
Lymphoproliferative	EBV	[103]
Multiple sclerosis	MS	[104]
Scarlet fever	SpeE, SpeA, SSA, SpeC	[105]
Sepsis	SpeJ, SMEZ	[78]
Severe acute hepatitis in children	SARS-CoV-2	[106]
Toxic shock syndrome	TSST-1/SEB/SEC	[107]
